# Self-Exfoliation of Flake Graphite for Bioinspired Compositing with Aramid Nanofiber toward Integration of Mechanical and Thermoconductive Properties

**DOI:** 10.1007/s40820-022-00919-0

**Published:** 2022-08-20

**Authors:** Limei Huang, Guang Xiao, Yunjing Wang, Hao Li, Yahong Zhou, Lei Jiang, Jianfeng Wang

**Affiliations:** 1 College of Materials Science and Engineering, Hunan University, Changsha, 410082 People’s Republic of China; 2 College of Chemistry and Chemical Engineering, Hunan University, Changsha, 410082 People’s Republic of China; 3 CAS Key Laboratory of Bio-Inspired Materials and Interface Sciences, Technical Institute of Physics and Chemistry Chinese, Academy of Sciences, Beijing, 100190 People’s Republic of China

**Keywords:** Graphene, Aramid nanofiber, Self-grinding exfoliation, Bioinspired structure, Functional material

## Abstract

**Highlights:**

- A self-grinding exfoliation strategy that depends on mutual shear friction between flake graphite particles is successfully developed to prepare pristine graphene with largely enhanced yield and productivity.
- Bioinspired assembly of pristine graphene nanosheets to an interconnected aramid nanofiber network is achieved by a continuous sol-gel-film transformation strategy and generates a flexible yet highly thermoconductive film.

**Abstract:**

Flexible yet highly thermoconductive materials are essential for the development of next-generation flexible electronic devices. Herein, we report a bioinspired nanostructured film with the integration of large ductility and high thermal conductivity based on self-exfoliated pristine graphene and three-dimensional aramid nanofiber network. A self-grinding strategy to directly exfoliate flake graphite into few-layer and few-defect pristine graphene is successfully developed through mutual shear friction between graphite particles, generating largely enhanced yield and productivity in comparison to normal liquid-based exfoliation strategies, such as ultrasonication, high-shear mixing and ball milling. Inspired by nacre, a new bioinspired layered structural design model containing three-dimensional nanofiber network is proposed and implemented with an interconnected aramid nanofiber network and high-loading graphene nanosheets by a developed continuous assembly strategy of sol–gel-film transformation. It is revealed that the bioinspired film not only exhibits nacre-like ductile deformation behavior by releasing the hidden length of curved aramid nanofibers, but also possesses good thermal transport ability by directionally conducting heat along pristine graphene nanosheets.

**Graphical abstract:**

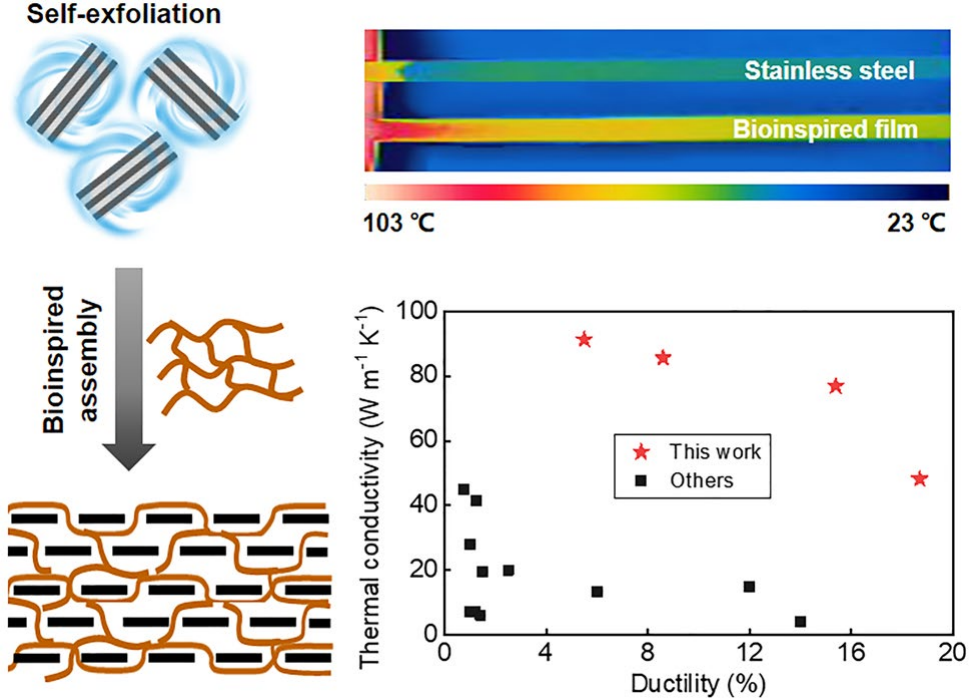

**Supplementary Information:**

The online version contains supplementary material available at 10.1007/s40820-022-00919-0.

## Introduction

Flexible yet highly thermoconductive materials are increasingly needed in electronic devices to dissipate the accumulated heat because of their high integration and miniaturization [1–6]. Hybridizing thermoconductive inorganic materials with flexible polymer materials is expected to integrate their respective advantages to obtain flexible yet thermoconductive materials [7–9]. It is proved theoretically and experimentally that graphene with *sp*^2^ carbon hybridization in two-dimensional honeycomb lattice has ultrahigh in-plane thermal conductivity (~ 5000 W m^−1^ K^−1^) and is a competitive candidate for the fabrication of thermoconductive composite films [10–13]. Graphene-based composites have made substantial progress in the past decade, and their thermal conductivity enhancement depends largely on graphene exfoliation and the co-assembly of graphene with polymers. For graphene exfoliation, liquid-based direct delamination strategies are widely used, such as ultrasonication [14, 15], high-shear mixing [16–18] and ball milling [19, 20]. The delamination is achieved by overcoming the van der Waals attraction within bulk graphite through the shear force generated between graphite and collapsed cavitation bubbles, mixing head, or grinding ball, avoiding the formation of basal-plane defects [21]. Nevertheless, only a small part of graphite is gradually converted into graphene, leaving a large amount of graphite residues. Careful analysis tells that the low yield and productivity are mainly related to the limited specific shear working area. For instance, the high-shear mixer used widely in the laboratory only provides a specific shear working area of a few square centimeters per gram of graphite, generating a low-concentration graphene dispersion with a yield of 0.1% and a production rate of 0.017 g h^−1^ L^−1^ [17]. A typical planetary ball mill can provide a specific shear working area from several square centimeters to a few tens of square centimeters per gram of graphite, dependent on the loading amount of grinding mediums, leading to a slightly improved yield and productivity [20]. High-yield, high-efficiency preparation of graphene from bulk graphite by liquid-based direct delamination remains a great challenge although it is critically important to develop thermoconductive composites toward practical application.

For the co-assembly of the exfoliated graphene and polymers, layered composite structure with high-loading, well-aligned graphene is one of the most effective structural patterns, which makes full use of the ultrahigh in-plane thermal conductivity of graphene nanosheets for directional heat conduction along orientation direction [22]. Solution-based assembly strategies that directly process graphene-polymer mixed solution into solid composites, such as evaporation induced self-assembly [23–25], vacuum-assisted filtration [26–29], ice templating [30–33] and doctor blading [34, 35], have been successfully developed to obtain well-ordered layered composite structure. The obtained composites are generally brittle, especially at high graphene loadings [36], because parallel graphene nanosheets confine the polymer chains in nanoscale-thick space and restrain the extension of coiled polymer chains under tensile stress [37]. Interestingly, natural nacre has a layered composite structure with 95% inorganic nanoplatelets, but can yield at a high tensile stress and then elongate plastically [38–40]. Microstructural observation reveals that the minor polymer phase not only contains crystalline chitin fibrils with a diameter of several nanometers, but also these fibrils are intertwined with each other, forming a robust three-dimensional network [38]. The network can homogenize stress and provide energy dissipation mechanism by the slippage and deformation of fibrils over a large volume because of their high interconnectivity. The three-dimensional chitin nanofibril network within the layered composite structure of natural nacre provides an inspiration for designing graphene-based thermoconductive composites with improved mechanical flexibility.

**Fig. 1:**
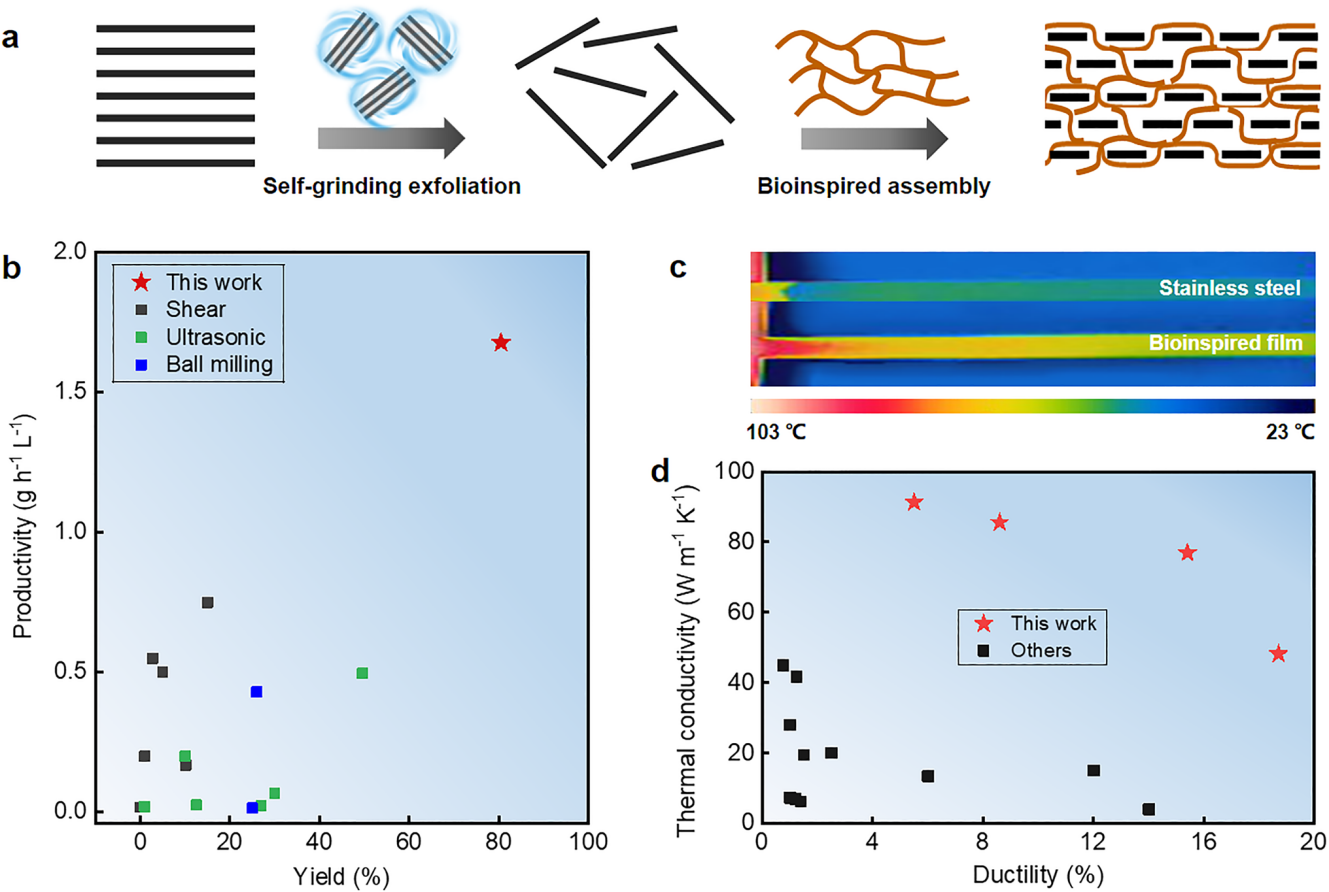
Flexible yet highly thermoconductive bioinspired film based on self-exfoliated pristine graphene and three-dimensional aramid nanofiber (ANF) network. **a** Schematics of pristine graphene exfoliation via a self-grinding strategy and bioinspired assembly with an interconnected aramid nanofiber network, forming a nacre-like layered composite structure. **b** Exceptional yield and productivity of graphene exfoliated by self-grinding and comparison with other liquid-based direct exfoliation approaches. The reference data are listed in Table S1. **c** A thermal image showing rapid heat transport on the bioinspired nanocomposite film (graphene loading 40%) and comparison with the metal alloy of stainless steel. **d** Thermal conductivity and ductility of the bioinspired nanocomposite film and careful comparison with other layered graphene-based composites. The relevant data are listed in Table S2

Herein, we propose a new liquid-based direct delamination strategy of self-grinding exfoliation that makes use of mutual shear friction between graphite particles to exfoliate graphene from flake graphite (Fig. 1a, left). The surface of graphite particles provides a substantially increased shear working area, which obviously improves exfoliation yield (80.5%) and efficiency (1.68 g h^−1^ L^−1^) (Fig. 1b). Inspired by natural nacre, we design a layered graphene-based composite structure containing a robust three-dimensional aramid nanofiber network (Fig. 1a, right). A continuous assembly strategy that processes graphene-aramid nanofiber mixed sol to hydrogel, then to solid film is developed to achieve the bioinspired structure. It is proved that the three-dimensional interconnected aramid nanofiber network well implements energy dissipation mechanism through fiber deformation and slippage, while high-loading, defect-few graphene nanosheets constitute a significant in-plane heat conduction path along their orientation direction, leading to well integration of thermal conductivity (48.2–91.3 W m^−1^ K^−1^) and post-yield ductility (5.5–18.6%) in the graphene-based composite film (Fig. 1c–d). These findings could offer innovative insights into structural design and preparation of flexible yet thermoconductive graphene-based composites for practical application.

## Experimental

### Materials

Flake graphite (CAS: 332461-12 KG, Sigma-Aldrich), commercial aramid microfiber (Kevlar 29, Dongguan SOVETL Co., Ltd), potassium ethoxide (≥ 95%, Sigma Aldrich), dimethyl sulfoxide (DMSO, CAS: 67–68-5, Tianjin HengXing Chemical Reagent Co., Ltd) and ZrO_2_ bead (0.5 mm in diameter, Saint-Gobain) were used as received.

### Self-Grinding Exfoliation of Graphene from Flake Graphite

Flake graphite was added into a grinding tank containing the solvent of DMSO. ZrO_2_ mirobeads with a diameter of 500 μm as grinding media were loaded into the tank, followed by grinding at a peripheral speed of 23 m s^−1^. Unless otherwise stated, the grinding time is fixed to be 24 h. The weight ratio of ZrO_2_ mirobeads to DMSO is fixed to be 5.3. In order to decrease the interparticle distance between flake graphite for self-grinding, initial graphite concentration is increased from 1 to 50 mg mL^−1^.

### Preparation of Graphene/Aramid Nanofiber Film

Graphene/aramid nanofiber film is prepared continuously by a sol–gel-film transformation process. Commercial aramid microfiber, potassium ethoxide and DMSO were mixed at a weight ratio of 2: 1.5: 96.5. The mixture was mechanically stirred for two days at 40 ℃, forming a viscous and dark red aramid nanofiber (ANF) solution. The ANF solution was mixed with the exfoliated graphene by mechanical stirring, forming a uniform sol. The weight ratio of graphene to ANF is controlled to be 40/60, 50/50, 60/40, and 70/30. The sol was extruded continuously from a die with a rectangular gap (8 × 0.75 mm^2^) into water for gelation. DMSO and potassium ethoxide were gradually diffused into water, generating a robust, black hydrogel. Finally, the hydrogel was dried to remove water at about 50 ℃, forming a ductile film with a thickness of about 30 μm.

### Characterization

Morphology characterizations were carried out in a field-emission scanning electron microscope (SEM, S-4800, Hitachi), transmission electron microscopy (TEM, JEM-3010, JEOL) and atomic force microscopy (AFM, Dimension Icon, Bruker). Raman spectra were taken in a Raman spectrometer (Alpha 300, Witec). X-ray photoelectron spectroscopy (XPS) was performed in an X-ray photoelectron spectroscopy excitation (ESCALAB 250Xi, Thermo Scientific). X-ray diffraction (XRD) spectra were collected in an x-ray diffractometer (MiniFlex, Rigaku). Rheometer test was carried out on a Haake Mars60, Thermo Fisher Scientific. Mechanical properties were measured on a Shimadzu AGS-X Tester. In tensile test, the gauge length and load speed are 5 mm and 1 mm min^−1^. Thermal conductivities were measured by a laser flash method (LFA 467 NanoFlash, Netzsch). Values of thermal conductivity were calculated from the equation *K* = *α* × *C*_p_ × *ρ*, where *K*, *α*, *C*_p_ and *ρ* represent thermal conductivity, thermal diffusivity coefficient, specific heat capacity and density, respectively. Specific heat capacity was measured with a differential scanning calorimetry (Q20, TA). Thermal diffusion coefficient in plane was measured using an LFA 467 NanoFlash. Density was obtained by the calculation of sample dimension and weight. High-temperature stabilities were evaluated by thermogravimetric analysis (TGA, STA 2500, Netzsch), and thermal mechanical analysis (TMA, Q400EM).

## Results and Discussion

### Self-Grinding Exfoliation of Graphene from Flake Graphite

**Fig. 2:**
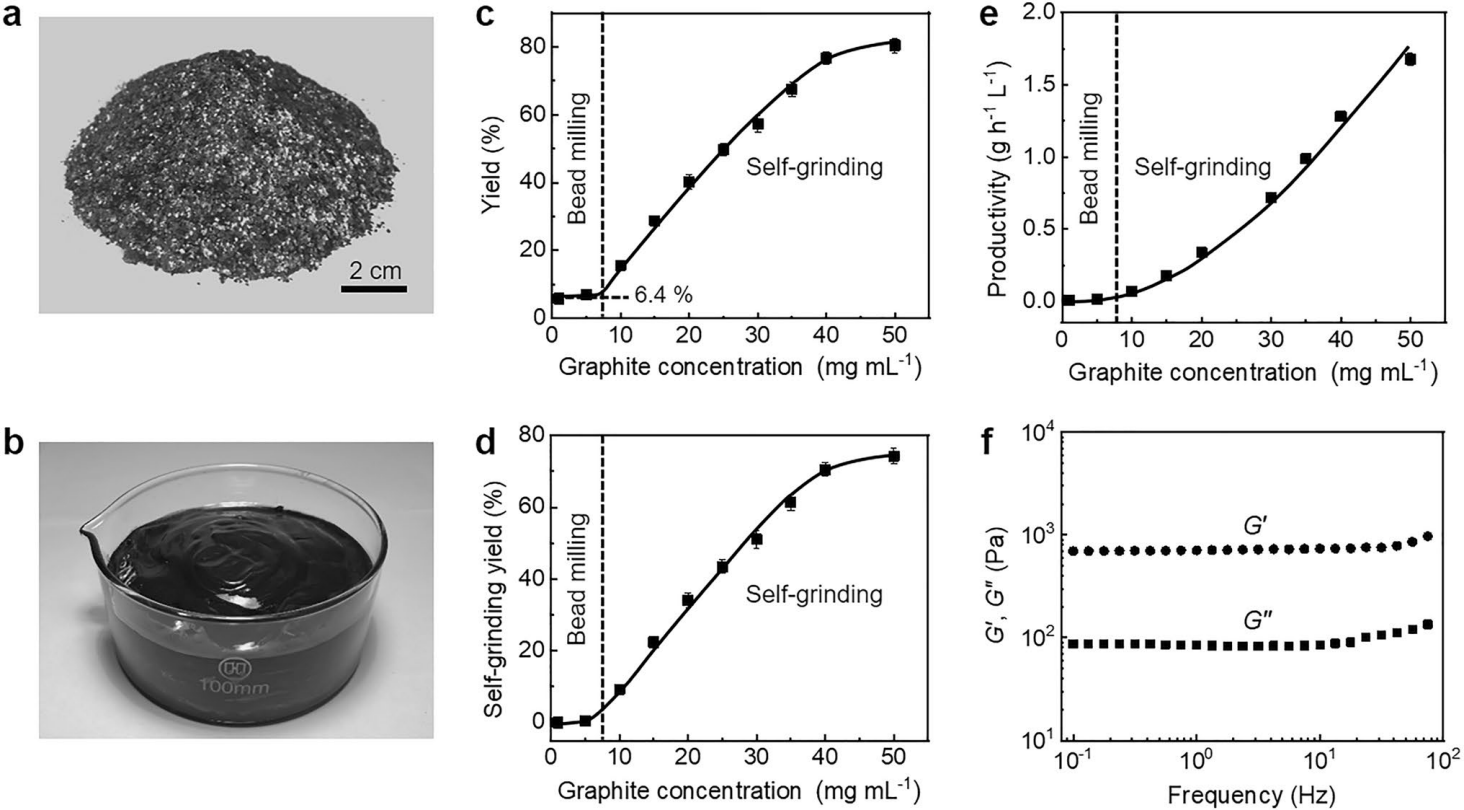
Dependence of self-grinding exfoliation on initial graphite concentration. **a** Flake graphite. **b** 1 kg graphene paste. **c** Yield change at an increasing graphite concentration. **d** Contribution of self-grinding to yield at an increasing graphite concentration. **e** Productivity change at an increasing graphite concentration. **f** Change of *G*′ and *G*″ with oscillation frequency at a constant strain of 1%

To implement the concept of self-grinding exfoliation, we design a microbead milling process which is characterized by high initial concentration of flake graphite (up to 50 mg mL^−1^) and small diameter of grinding beads (500 μm). At high initial concentration, graphite particles would generate a decreased interparticle distance and even contact physically, which enables their mutual shear friction. To drive the rotation of graphite particles at such high initial concentration, small-size grinding beads are used, which help to form stable turbulent flow, homogenize the whole mixed system and improve the exfoliation yield and productivity during grinding (Fig. S1). It is different from previously used ball milling process that can only exfoliate graphite at a low concentration by using large balls with a typical diameter from several millimeters to centimeters [41, 42]. In appearance, the coarse mixture with large-size flake graphite becomes a viscous, smooth graphene paste after grinding for a prolonged time (Fig. 2a–b and S2).

The dependence of self-grinding exfoliation on initial graphite concentration is carefully investigated by measuring the yield of graphene. As shown in Fig. 2c, when graphite concentration is less than 10 mg mL^−1^, graphene yield is stabilized at a low value of about 6.4%, similar with the results by previous liquid-based direct exfoliation [43]. It implies that the shear action only through the friction between microbeads and graphite particles is not enough to delaminate graphite sufficiently. This is further confirmed by the fact that increasing the loading weight of grinding microbeads in a wide range only generates a slight increase in yield (Fig. S3). Strikingly, a sharp increase occurs once the initial graphite concentration increases from 10 to 40 mg mL^−1^. Afterward, the yield levels off and is up to 80.5% at an initial concentration of 50 mg mL^−1^. It indicates that self-grinding between graphite particles at high concentration plays a dominant role in the exfoliation. We assume that the contribution of microbead-graphite grinding to yield is the same as that in the low initial concentration range, and the contribution of graphite-graphite grinding to yield is calculated, as shown in Fig. 2d. It is clearly seen that the graphite-graphite grinding becomes more and more important at an increasing initial graphite concentration and finally contributes to an increase of 74.1% in yield. Due to the increased yield, the self-grinding leads to a sharp improvement in the productivity of graphene, as shown in Fig. 2e. A 25-fold improvement from 0.065 to 1.7 g h^−1^ L^−1^ is presented when the initial graphite concentration increases from 10 to 50 mg mL^−1^.

Rheological test is carried out to prove that graphite particles at the high concentration form a jammed network for self-grinding exfoliation. Frequency sweep shows that the storage modulus of the grinded mixture is an order of magnitude higher than its loss modulus over the whole frequency ranging from 0.1 to 100 Hz, indicating that elastic response is predominant (Fig. 2f). It reveals that the grinded particles at a concentration of 50 mg mL^−1^ form a quasi-solid network due to steric hindrance [44, 45]. Strain sweep shows that storage modulus surpasses loss modulus at small shear strain, while the storage modulus falls below the loss modulus at an increasing shear strain above 8%, indicating that the jammed network starts to collapse with relative slippage between graphite particles (Fig. S4a). Steady rheological test shows a sharp decrease in viscosity by three orders of magnitude at an increasing shear rate from 0.1 to 1000 s^−1^ (Fig. S4b). The remarkable decrease is visualized by manually stirring the grinded mixture with a glass rod (Fig. S4c). It is converted from a viscosity paste into a flow state. The prominent shear thinning behavior facilitates the formation of stable turbulent flow during microbead milling to homogenize the whole grinded mixture [46].

### Morphology and Structure of Exfoliated Graphene

**Fig. 3:**
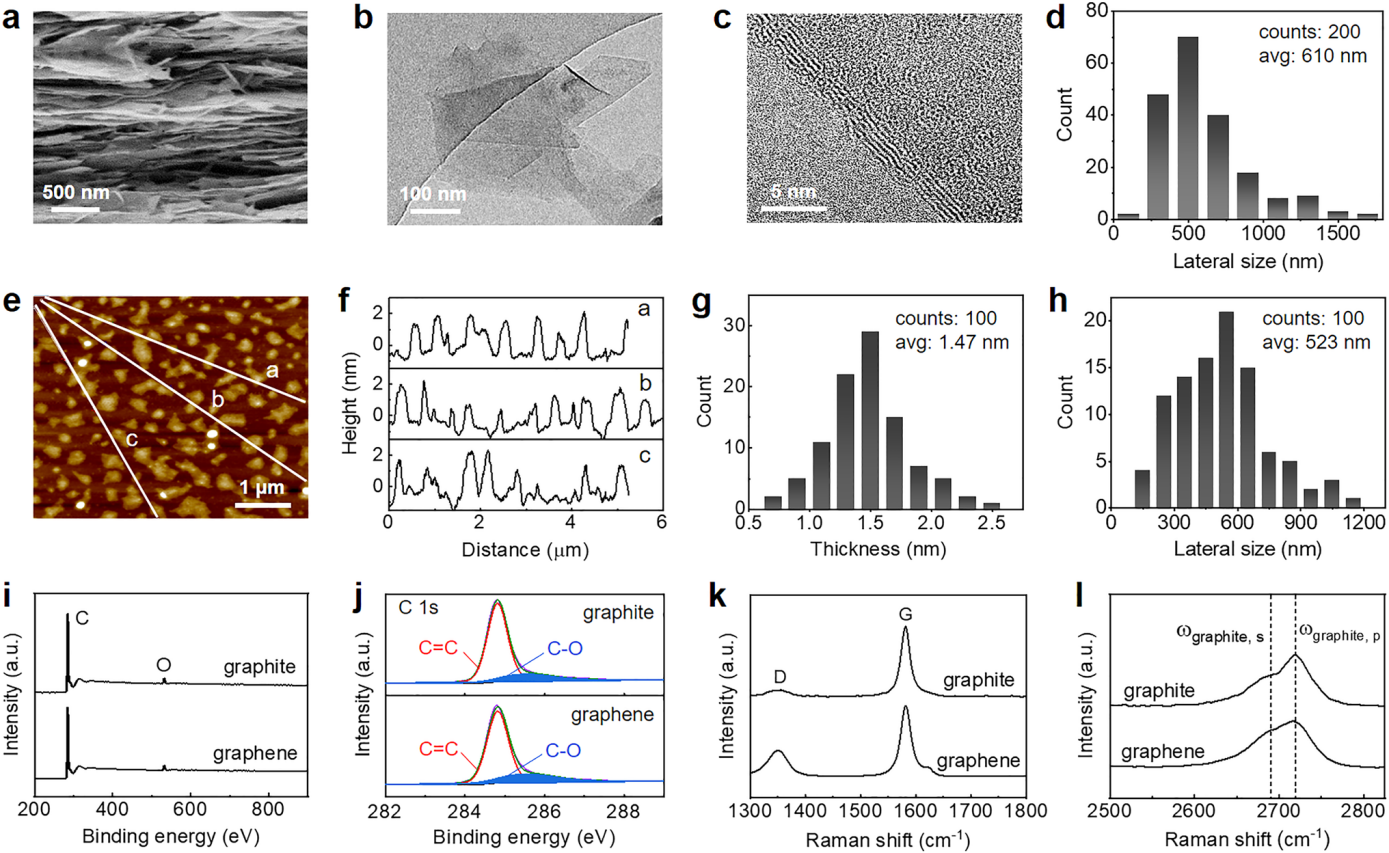
Characterization of the exfoliated graphene. **a** SEM image. **b** Low-magnification TEM image. **c** Representative high-resolution TEM image. **d** Lateral size distribution extracted from TEM images. **e** AFM image. **f**–**h** Height profiles, thickness and lateral size distribution extracted from AFM images. **i** XPS spectra. **j** Deconvolution of C 1*s* peak. **k**–**l** Raman spectra

Morphology and size of the exfoliated graphene were characterized by SEM, TEM and AFM. SEM images exhibit a sheet morphology with irregular geometry (Fig. 3a and S5). Low-magnification TEM images show that the sheets are transparent, indicating a thin thickness (Fig. 3b). Representative high-resolution images at the edge of sheets show five parallel lines with 0.34 nm distance between two adjacent lines, consistent with theoretical *d*-spacing of few-layer graphene (Fig. 3c). The lateral size distribution of the sheets obtained by statistical analysis from many low-magnification TEM images is in the range from 180 nm to 1.71 um with an average value of 610 nm, comparable to the size of graphene exfoliated by ultrasonic wave (Fig. 3d). AFM images also prove that the exfoliated graphene presents thin sheet morphology, consistent with SEM and TEM observations (Fig. 3e). The height profiles were extracted carefully from 100 sheets to determine the lateral size and thickness of graphene, as shown in Fig. 3f–h. It is seen that their lateral size ranges from 152 to 1182 nm with an average value of 523 nm, consistent with the statistical results from TEM images. Their thickness is in the range from 0.7 to 2.5 nm with an average value of 1.47 nm, indicating the exfoliated graphene has an average thickness of 4.4 atomic layers.

The structure of the exfoliated graphene was characterized by XRD, XPS, and Raman spectra. As expected, XRD curves show a substantially decreased intensity and increased width of diffraction peak at 26.5° in comparison to raw flake graphite because of delamination along basal plane direction (Fig. S6). XPS spectra show that the graphene contains the dominant carbon element and a little oxygen element (Fig. 3i). The oxygen content is only 4.14 atom%, as low as raw flake graphite (3.75 atom%). Deconvolution of C 1s peak reveals that *sp*^2^-hybridized carbon arising from honeycomb lattice in the basal plane of graphene is predominant, and the peak area of C–O has no obvious change, compared with raw flake graphite (Fig. 3j). These comparisons indicate that the self-grinding exfoliation is a pure physical process without occurrence of oxidation reaction, thus avoiding the introduction of heteroatom on the basal plane of graphene. Raman spectra clearly show a predominant *G* peak at 1580 cm^−1^ and a minor *D* peak at 1325 cm^−1^, corresponding to *sp*^2^-hybridized aromatic carbon and *sp*^3^-hybridized carbon, respectively (Fig. 3k) [21]. Compared with raw flake graphite, the intensity ratio of D peak to G peak is slightly increased (0.10 versus 0.33), indicating that the exfoliation process introduces a few defects. The increased intensity ratio is similar to the graphene prepared by other liquid-based direct delamination approaches (ultrasonication, high-shear mixing and ball milling) that only generates a few defects at the edge of graphene. The increased intensity ratio is probably associated with the substantial decrease in lateral size from several hundreds of micrometers to several hundreds of nanometers during exfoliating flake graphite into graphene. Another change is the shape of 2D peak at 2718 cm^−1^, which can be used to quantitatively estimate the average number of graphene layers by using the following empirical formula [17]:

1 $$N_{G} = 10^{{0.84M + 0.45M^{2} }}$$

Here *M* is a dimensionless parameter that describes the shape change of 2D peak. It can be calculated by the following formula:

2 $$M = \frac{{{{I_{{{\text{graphene}},p}} } \mathord{\left/ {\vphantom {{I_{{{\text{graphene}},p}} } {I_{{{\text{graphene}},s}} }}} \right. \kern-\nulldelimiterspace} {I_{{{\text{graphene}},s}} }}}}{{{{I_{{{\text{graphene}},p}} } \mathord{\left/ {\vphantom {{I_{{{\text{graphene}},p}} } {I_{{{\text{graphene}},s}} }}} \right. \kern-\nulldelimiterspace} {I_{{{\text{graphene}},s}} }}}}$$

Here *I*_graphite, p_ and *I*_graphite, s_ are the intensities of 2D peak and shoulder of graphite, while *I*_graphene, p_, and *I*_graphene, s_ are the intensities of 2D peak and shoulder of graphene at the same location as graphite (Fig. 3l). As a result, the average number of layers per graphene is calculated to be 6.8, close to the value obtained by statistics from AFM images. In a word, the self-grinding strategy successfully exfoliates few-layer graphene nanosheets with a few defects.

### Continuous Assembly and Microstructure of Graphene/Aramid Nanofiber Film

**Fig. 4:**
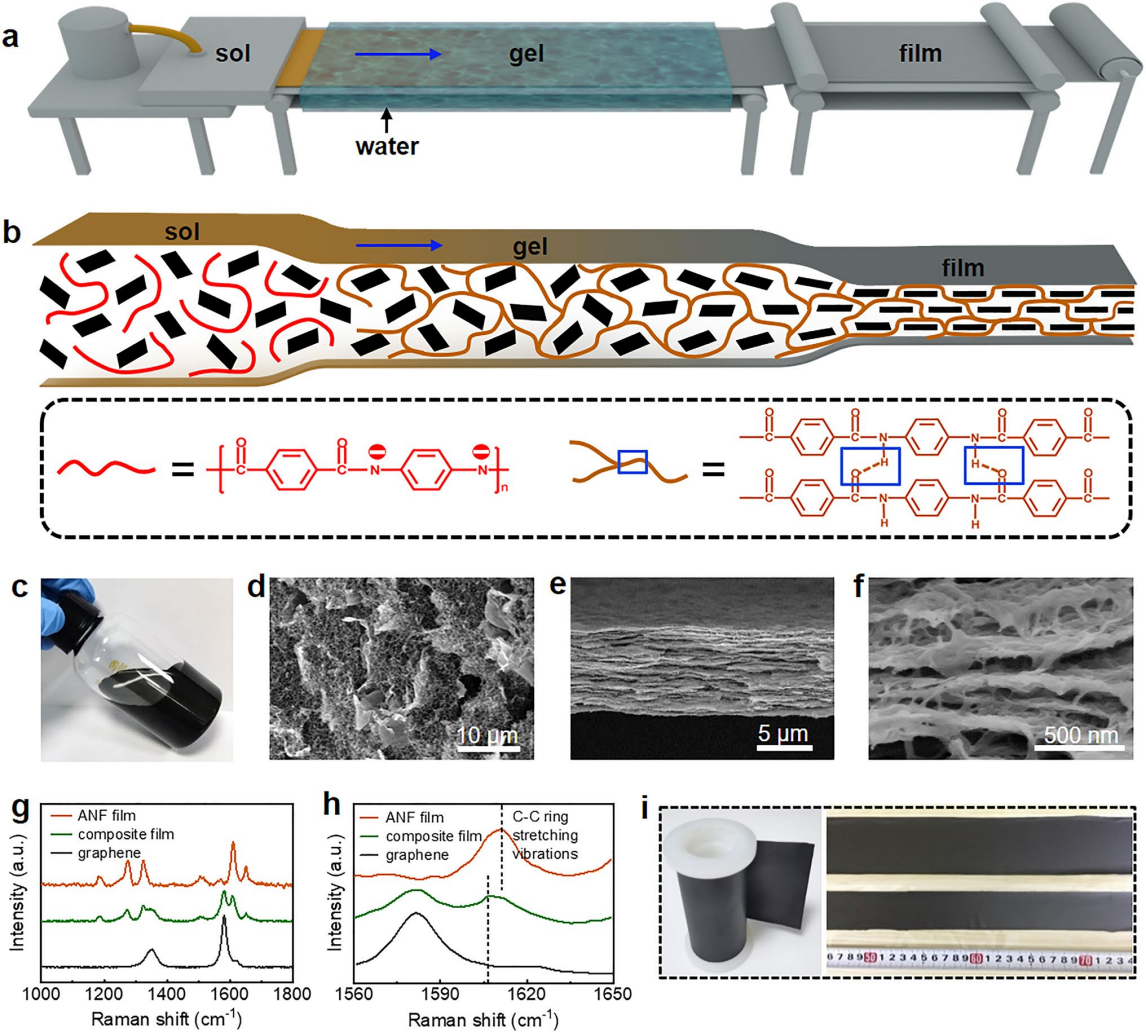
Fabrication and microstructure of bioinspired graphene/aramid nanofiber film. **a** Illustration of the setup for sol–gel-film transformation. **b** Schematics of the microstructure evolution during sol–gel-film transformation. **c** Graphene-aramid nanofiber sol. **d** SEM image of graphene/aramid nanofiber hydrogel. **e**–**f** SEM images of graphene/aramid nanofiber film. **g**–**h** Raman spectra of graphene/aramid nanofiber film and comparison with graphene and ANF film. The characteristic peak located in 1610.8 cm^−1^ presents C–C ring stretching vibrations of poly(*p*-phenylene terephthalamide) chains. **i** Continuous composite film with adjustable width

Based on the exfoliated graphene and aramid nanofiber, we develop a continuous assembly strategy of sol–gel-film transformation to mimic the unique microstructure of natural nacre for improving the toughness of graphene-based thermoconductive composite materials (Fig. 4a). Aramid nanofiber is chosen because of its small diameter (5–15 nm), high crystallinity and excellent strength, similar to the chitin nanofibril in natural nacre [47]. The transformation from graphene-aramid nanofiber sol in DMSO to hydrogel is to pre-organize aramid nanofibers into a three-dimensional network to increase the interconnectivity between nanofibers (Fig. 4b). In this process, curved aramid nanofibers with the removal of proton from amide groups by strong alkali in DMSO is re-protonated by water and entangled each other, due to a decreased electrostatic repulsion and increased hydrogen bond attraction between nanofibers [48]. Subsequent transformation from hydrogel to solid film by drying is to orient graphene nanosheets, similar to the evaporation-induced self-assembly process driven by capillary force during water removal.

The assembly process to form nacre-like layered composite structure is confirmed by SEM observation of its structure evolution, as shown in Fig. 4c–f. The hydrogel consists of a porous nanofiber network containing many uniformly distributed graphene nanosheets (Figs. 4d and S7). The solid film presents a well-defined layered structure with well-oriented graphene nanosheets along film plane (Fig. 4e). Enlarged view shows that the layered composite structure contains a three-dimensional nanofiber network in which the curved nanofibers are interconnected in the thickness and plane directions of film, similar to the chitin nanofibril network in natural nacre (Figs. 4f and S8). The interfacial interaction between graphene sheets and aramid nanofiber network is investigated by Raman spectra, as shown in Fig. 4g. Both the characteristic peaks assigned to the Raman scattering from aramid nanofiber (C–C ring stretching vibrations: 1186, 1275, 1507, and 1610 cm^−1^, C–H in plane bending vibration: 1325 cm^−1^, C=O stretching vibration: 1651 cm^−1^) and graphene (*sp*^3^-hybridized carbon: 1325 cm^−1^, *sp*^2^-hybridized carbon: 1580 cm^−1^) are present [49]. The change is that the characteristic peak of aromatic ring of aramid molecules is red-shifted from 1610.8 to 1606.3 cm^−1^ in comparison to pure ANF (Fig. 4h), revealing the existence of π-π interaction between the nanofiber network and the nanosheets [47, 50]. The assembly strategy of sol–gel-film transformation to obtain nacre-like structure is convenient and can be used to continuously prepare composite films with different graphene loadings by altering the ratio of graphene to aramid nanofiber in the sol, as well as composite films with different thicknesses and widths by adjusting the gap size of the die used for sol extrusion (Fig. 4i).

### Integrated Mechanical and Thermal Properties of Graphene/Aramid Nanofiber Film

**Fig. 5:**
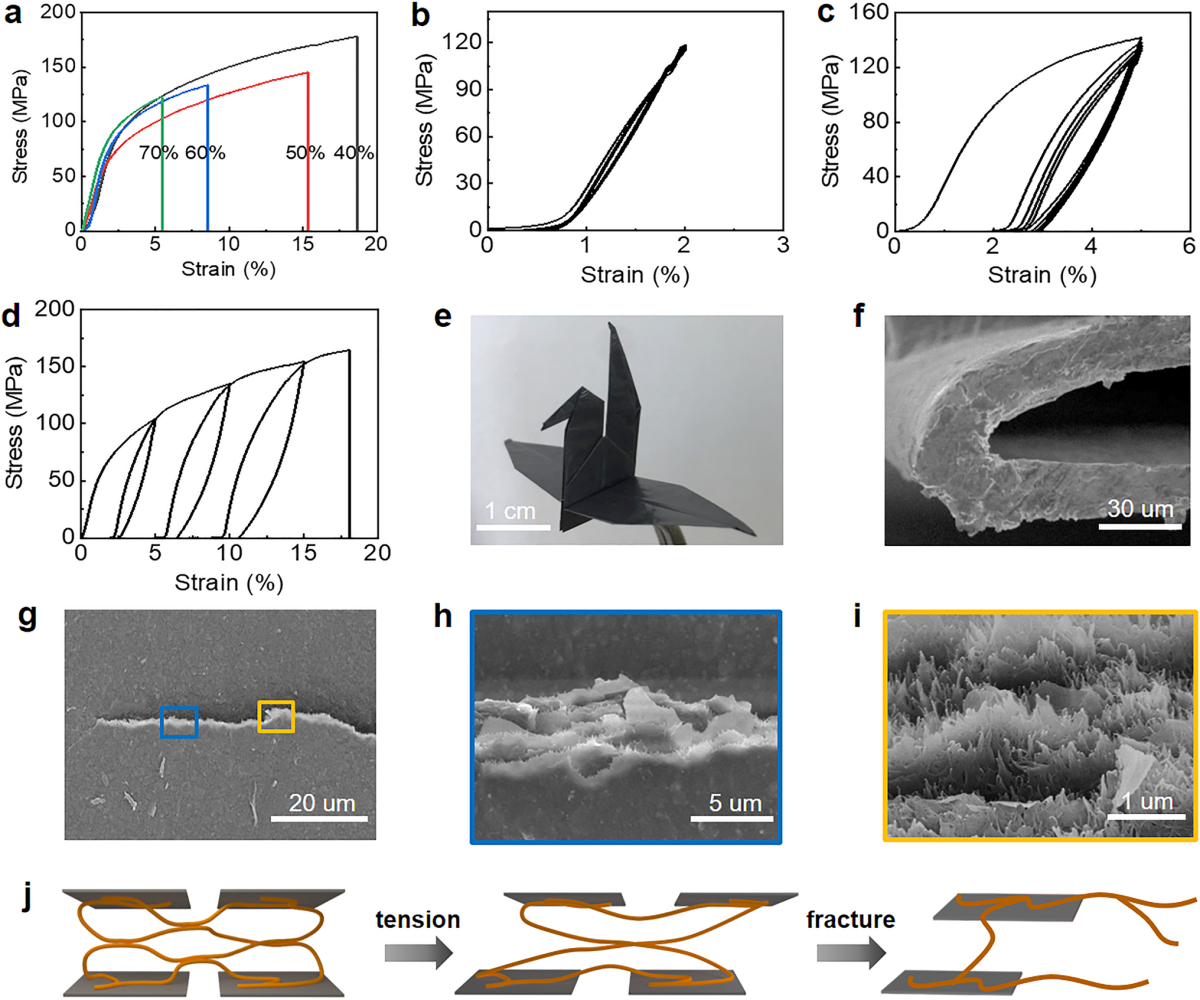
Deformation of bioinspired graphene/aramid nanofiber film. **a** Tensile stress–strain curves of the film with different graphene loadings. **b** Cyclic tensile curve at a small strain of 2% for 5 cycles. **c** Cyclic tensile curve at a relatively large strain of 5% for 5 cycles. **d** Cyclic tensile curve at an increasing strain of 4%, 10%, and 15%. **e** An origami crane folded from a piece of composite film with a size of 8 × 8 cm^2^. **f** SEM image of cross section after repeated folding for 50 times. **g**-**i** SEM images of a propagated crack. **j** A proposed tensile deformation model

The graphene/aramid nanofiber films with different graphene loadings assembled by the sol–gel-film transformation strategy were measured in tensile mode to investigate their deformation behavior (Fig. S9). And their representative stress–strain curves are shown in Fig. 5a. Overall, although containing a high graphene loading (> 40%), they exhibit nacre-like deformation behavior that progressive yield and subsequent plastic elongation accompanying with strain hardening occurs after linearly elastic deformation. As expected, the ultimate strain at break depends on the graphene loading and changes in the range of 5.5% and 18.6%. The ductile deformation behavior is in stark contrast to other layered graphene/polymer and graphene/nanofiber films with high pristine graphene loadings that facture brittlely after linearly elastic deformation [36, 51], indicating the importance of three-dimensional interconnectivity of aramid nanofibers.

The nacre-like deformation behavior is further proved by cyclic tension test. Cyclic loading–unloading at a small strain in the elastic deformation region exhibits negligible hysteresis, while obvious hysteresis with large loops is generated by cyclic loading–unloading at a strain above the yielding strain (Fig. 5b–c). It implies that the nanoscale building blocks in the layered composite structure start to slide after yielding, generating prominent energy dissipation. Cyclic loading–unloading at an increasing strain enlarges the area of hysteresis loops (Fig. 5d), suggesting that the building blocks continue to slide to a greater extent. The intensive sliding is proved by crack observation of a loaded notched sample, as shown in Fig. 5g. Enlarged view of the tortuous crack shows that both graphene nanosheets and aramid nanofibers are pulled out toward the tensile direction (Fig. 5h–i).

A simplified tensile deformation model is illustrated to understand the relationship between the nacre-like microstructure and its tensile property, as shown in Fig. 5j. With an increasing tensile loading, the hydrogen bonding between aramid nanofibers that acts as sacrificial bond would be broken, leading to the macroscopic yield in the tensile stress–strain curve. Afterward, the curved aramid nanofibers straighten, slide with graphene nanosheet together and release their hidden length, corresponding to the macroscopic large plastic elongation in the tensile stress–strain curve. After the hidden length is exhausted, the nanofibers are pulled out with the nanosheets together, thus leading to large strain-to-failure and high toughness. The ductile graphene/aramid nanofiber film has good fold endurance and is easy to be folded into complex paper cranes without any damage (Fig. 5e). SEM observation shows that the film does not generate any microcrack after folding repeatedly for 50 cycles (Fig. 5f).

**Fig. 6:**
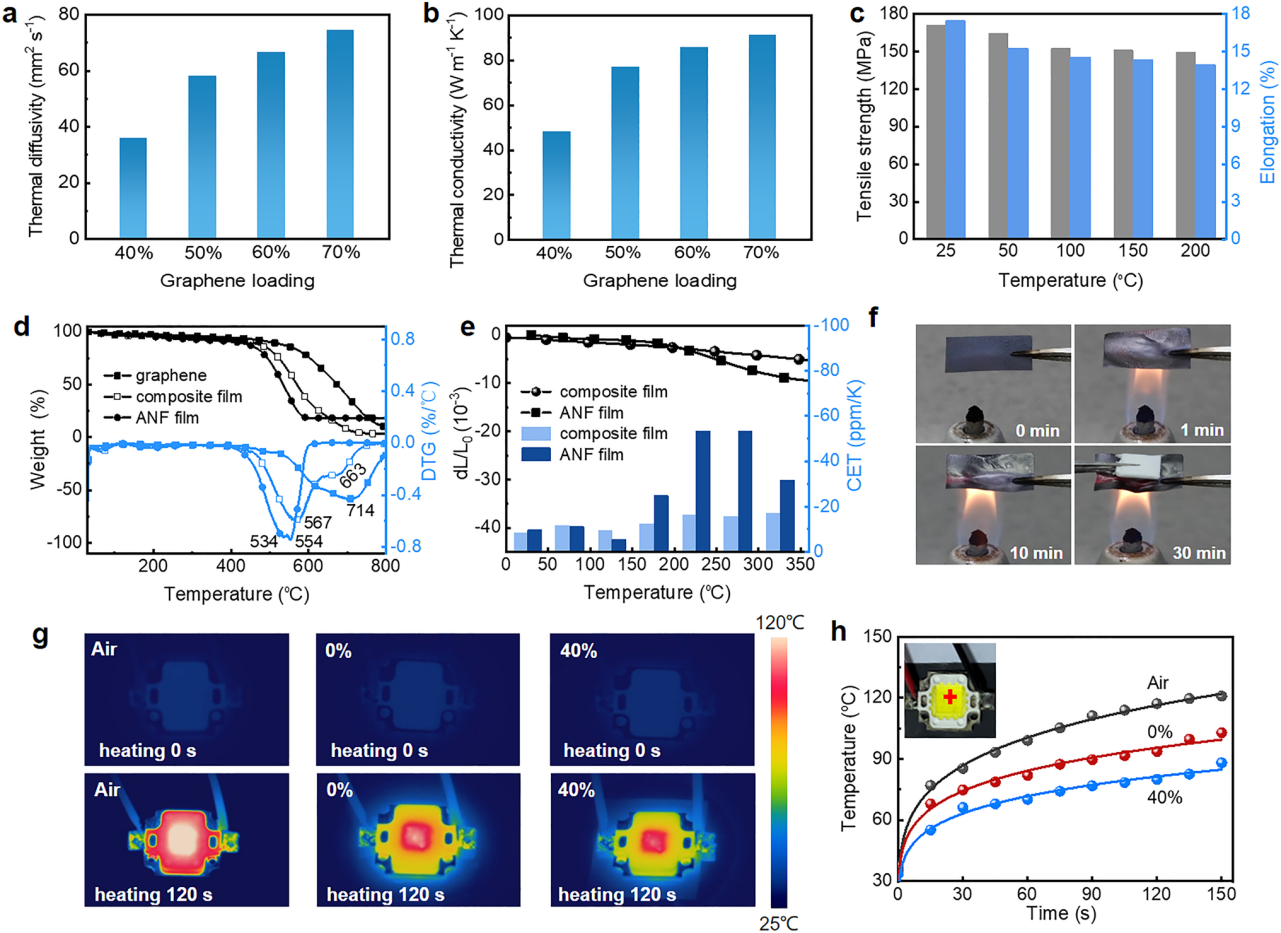
Thermal conduction and thermal stability of bioinspired graphene/aramid nanofiber film. **a** In-plane thermal diffusivity of the film with an increasing graphene loading. **b** In-plane thermal conductivity. **c** Tensile strength and elongation of composite film (40%) after heat treatment at different temperatures. **d** TGA and DTG curves of graphene, ANF film and composite film (40%). **e** d*L*/*L*_0_ and coefficients of thermal expansion (CTE) of composite film (40%) and ANF film at different temperatures. **f** Photographs of composite film (40%) before and during burning, showing nonflammability. **g** Comparison of infrared thermal images of air, aramid nanofiber (0%) and composite film (40%) as LED heat dissipating substrate. **h** Temperature evolution curves of hot spot at cross-point (inset) with working time

Besides mechanical deformation, thermal conduction of the graphene/aramid nanofiber films with different graphene loadings was evaluated by a laser flash method. As expected, their in-plane thermal diffusivity depends on graphene loading and ranges from 35.8 to 74.6 mm^2^ s^−1^ with the increased loading from 40 to 70% (Fig. 6a). Their in-plane thermal conductivity that are calculated based on the thermal diffusivity, specific heat capacity and density also increases monotonically with graphene loading and is in the range of 48.2 and 91.3 W m^−1^ K^−1^ (Fig. 6b). These values are higher than those of other pristine graphene/polymer composites with randomly oriented structure and reduced graphene oxide/polymer composites with well-oriented layered structure because of both few defects and well orientation of graphene nanosheets in the high-loading composite films (Table S2). Thermal conduction of the graphene/aramid nanofiber films is anisotropic. Their through-plane thermal conductivity is in the range of 0.22 and 0.44 W m^−1^ K^−1^, lower than the in-plane values because of horizontal orientation of graphene nanosheets (Fig. S10). Moreover, the composite film with 40% loading of graphene exhibits good thermal stability that the tensile strength and elongation remains about 150 MPa and 14% even at 200 ℃ (Fig. 6c). This stability is ascribed to the high-temperature durability of chemical components of composite film, that is, maximum decomposition temperature of aramid nanofiber and graphene are 554 and 714 ℃, respectively (Fig. 6d). It is worth noting that its maximum decomposition temperature of the composite film increases by 13 ℃, compared to ANF film, indicating interfacial interaction gives rise to higher thermal stability. This result is further confirmed by TMA test for dimensional change and coefficients of thermal expansion of the composite film and ANF film in the temperature range of 0 to 350 ℃ (Fig. 6e). More steady dimensional change and lower coefficients of thermal expansion of the composite film from − 8.4 to − 17.13 ppm up to 350 ℃ can efficiently avoid thermal failure arising from bad contact. Given that nonflammability of the components, we measured its flame retardancy as shown in Fig. 6f. After burning for 30 min, a piece of normal paper above the composite film is nonflammable due to exemplary flame-resistance capacity of the composite film.

The good heat conduction ability is visualized by placing the graphene/aramid nanofiber films with different graphene loadings under a high-power LED lamp as heat dissipating substrate for infrared thermal imaging (Fig. 6g). It is seen that the temperature for composite film with 40% graphene rises more slowly than that for air and composite film with 0% graphene (0.99 W m^−1^ K^−1^) after heating for 120 s. The temperature evolution curves of hot spot at cross-point with working time are displayed in Fig. 6h. The temperature drop depends on graphene loading in the film, and a significant decrease of 13.7 ℃ is caused by the film containing 40% graphene compared with that of 0% when heating for 120 s. The improved thermal conduction ability and good thermal stability along with superb flexibility enables the bioinspired film to act as heat dissipation materials in flexible electronic devices.

## Conclusions

A self-grinding exfoliation strategy is developed successfully through a designed microbead milling of high-concentration flake graphite to prepare pristine graphene with sharply increased yield and productivity. The obtained graphene nanosheets have few atomic layers in thickness, several hundreds of nanometers in lateral size and few defects without introduction of heteroatoms. A sol–gel-film transformation strategy is further developed to continuously assembly the graphene nanosheets and aramid nanofibers into nacre-like layered composite structure with a three-dimensional interconnected nanofiber network. The bioinspired film simultaneously exhibits nacre-like deformation behavior by releasing the hidden length of curved nanofibers and good thermal transport ability by directionally conducting heat along pristine graphene nanosheets, leading to the integration of large ductility and high thermal conductivity. The integrated properties make the composite film potential for heat management in flexible electronic devices.

## Supplementary Information

Below is the link to the electronic supplementary material.

Supplementary file1 (PDF 709 kb)
