# Supplementary material for: Self-Exfoliation of Flake Graphite for Bioinspired Compositing with Aramid Nanofiber toward Integration of Mechanical and Thermoconductive Properties
Source: Nanomicro Lett. 2022 Aug 20;14:168. doi: 10.1007/s40820-022-00919-0 (PMC9392675; doi:10.1007/s40820-022-00919-0)
Supplement: Supplementary file 1 — Supplementary file1 (PDF 709 kb) [file 40820_2022_919_MOESM1_ESM.pdf]

Supporting Information for

Self-Exfoliation of Flake Graphite for Bioinspired Compositing with Aramid Nanofiber toward Integration of Mechanical and Thermoconductive Properties

Limei Huang[1, #], Guang Xiao[1, #], Yunjing Wang[1], Hao Li[2], Yahong Zhou[3], Lei Jiang[1, 3] and Jianfeng Wang[1, *]

[1]College of Materials Science and Engineering, Hunan University, Changsha 410082, P. R. China

[2]College of Chemistry and Chemical Engineering, Hunan University, Changsha 410082, P. R. China

[3]CAS Key Laboratory of Bio-Inspired Materials and Interface Sciences, Technical Institute of Physics and Chemistry Chinese, Academy of Sciences, Beijing 100190, P. R. China

[#]Limei Huang and Guang Xiao contributed equally to this work.

*Corresponding author. E-mail: wangjianfeng@hnu.edu.cn (Jianfeng Wang)

Supplementary Figures and Tables

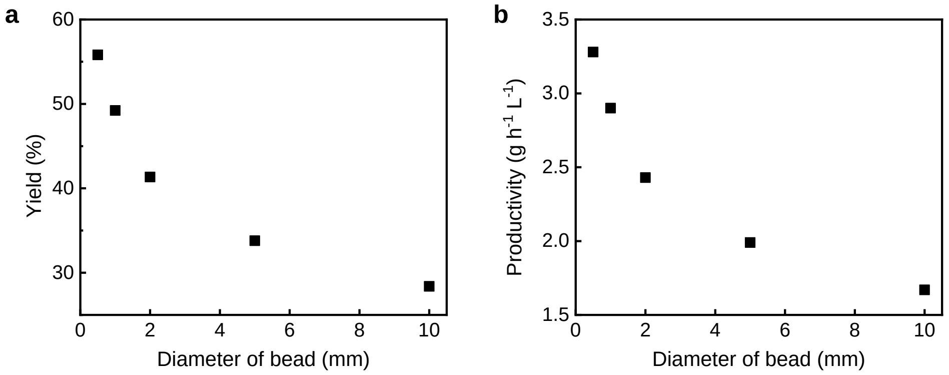

Fig. S1 Plots of graphene yield and productivity as a function of diameter of bead after the grinding of flake graphite with an initial concentration of 50 mg mL^{-1} for 17 h. **a** Change of graphene yield with the diameter of ZrO_2 bead. **b** Change of graphene productivity with the diameter of ZrO_2 bead. The results show that graphene yield is improved from 28.39% to 55.81%, and its productivity is improved from 1.67 to 3.28 g h^{-1} L^{-1} when the bead diameter decreases from 10 to 0.5 mm

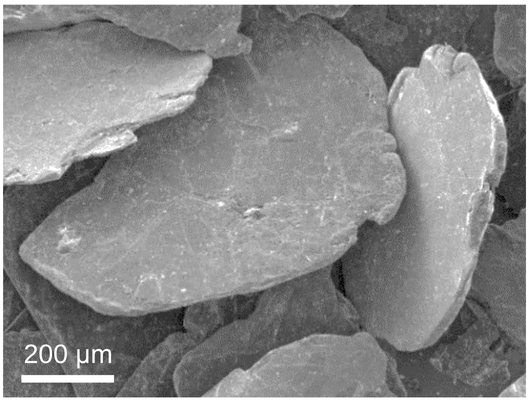

Fig. S2 SEM image of the used flake graphite

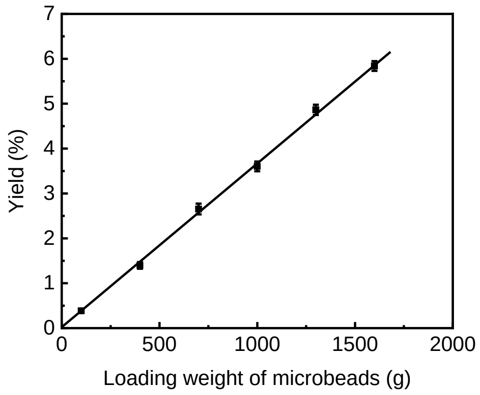

Fig. S3 Influence of microbead weight on the yield of graphene. The initial graphite concentration is fixed to be 1 mg mL^{-1}

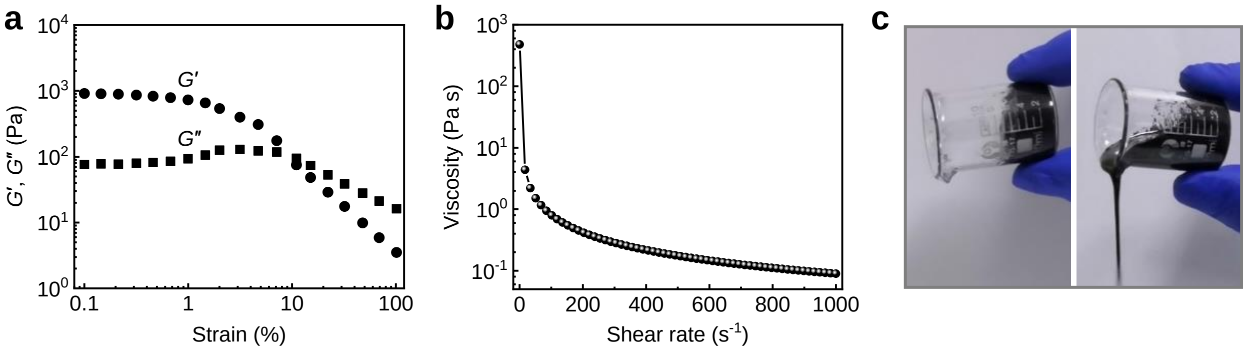

Fig. S4 Rheological behavior of the grinded mixture. **a** Change of storage modulus (G′) and loss modulus (G″) with oscillation strain at a constant frequency of 1 HZ. **b** Viscosity-shear rate curve. **c** Digital photos before (left) and after stirring with a glass rod (right)

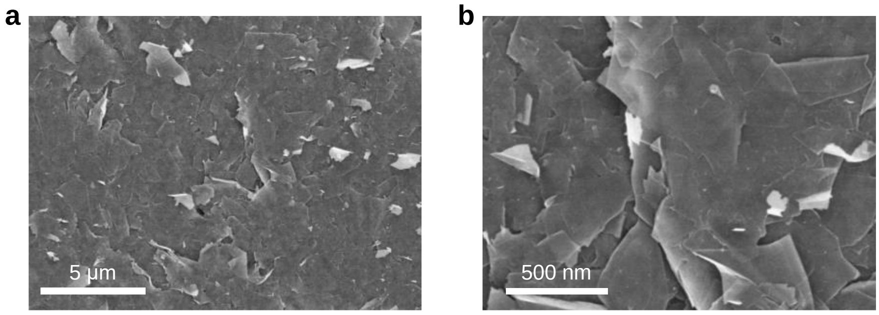

Fig. S5 SEM images of the exfoliated graphene nanosheets. **a** Low magnification. **b** High magnification

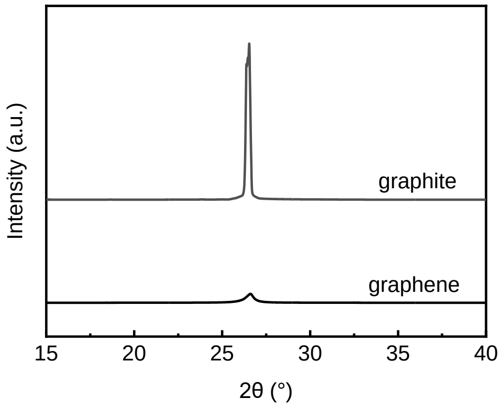

Fig. S6 XRD curves of graphite and the exfoliated graphene

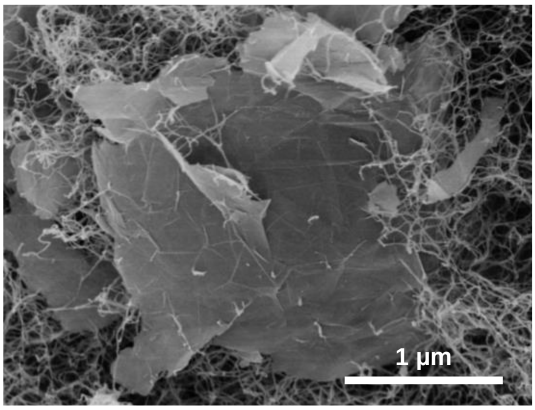

Fig. S7 Enlarged SEM image of graphene/aramid nanofiber hydrogel

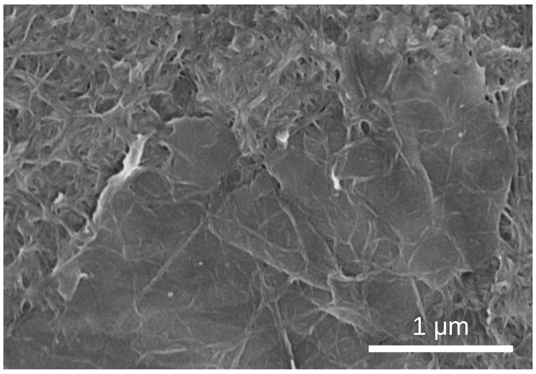

Fig. S8 SEM image of the surface of graphene/aramid nanofiber film, showing that the nanosheets are covered by aramid nanofiber network

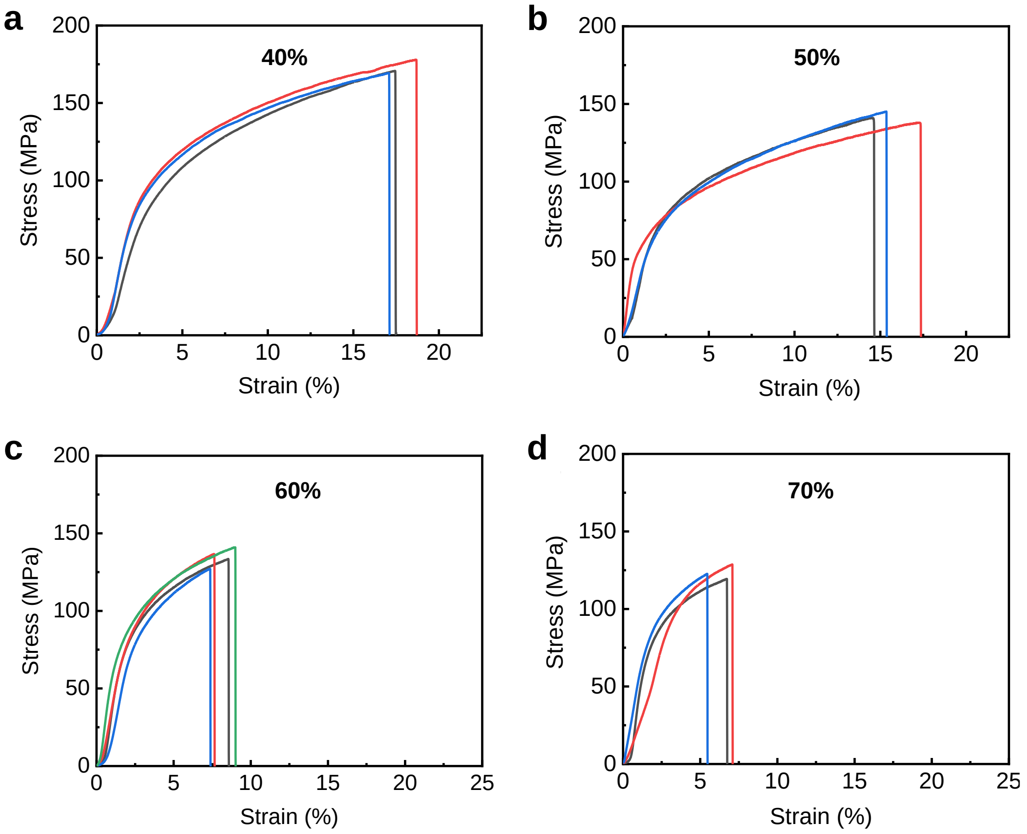

Fig. S9 Tensile stress-strain curves of graphene/aramid nanofiber films with an increasing graphene loading. **a** Graphene loading of 40%. **b** Graphene loading of 50%. **c** Graphene loading of 60%. **d** Graphene loading of 70%

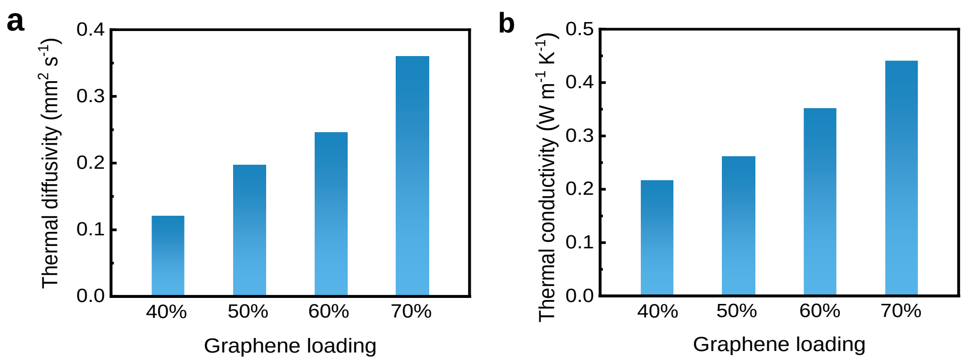

Fig. S10 a Through-plane thermal diffusivity of the composite film with an increasing graphene loading. **b** Through-plane thermal conductivity

Table S1 Comparison of yield and productivity of graphene exfoliated by self-grinding with other liquid-based direct exfoliation approaches

Strategy	Concentration (mg mL^{-1})	Yield (%)	Productivity (g h^{-1} L^{-1})	I_D/I_G	C/O ratio	Refs.
Shear	1.01	10.1	0.168	–	–	[S1]
	1	1	0.2	0.39	14.6	[S2]
	1.5	15	0.75	0.54	24.64	[S3]
	0.07	0.1	0.017	0.18	–	[S4]
	1.1	2.75	0.550	0.1-0.3	–	[S5]
	0.25	5	0.500	0.14	–	[S6]
	6	30	–	0.5	–	[S7]
	0.31	3.1	–	1.4	–	[S8]
	1.8	18	–	0.1-0.4	–	[S9]
Ultrasonic	0.05	12.5	0.025	0.47	–	[S10]
	0.54	27	0.023	0.28	–	[S11]
	0.2	10	0.200	–	–	[S12]
	1.5	30	0.066	0.38-0.45	9.2	[S13]
	0.992	49.6	0.496	–	–	[S14]
	0.01	1	0.02	–	–	[S15]
Ball milling	–	40	–	0.2	21.2	[S16]
	2.6	26	0.430	0.25	–	[S17]
	0.175	25	0.014	0.38	–	[S18]
	1	–	–	0.55	7.06	[S19]
**Self-grinding**	**50**	**80.5**	**1.68**	**0.33**	**23.15**	**This work**

Table S2 Comparison of mechanical and thermal conduction properties of the bioinspired graphene/aramid nanofiber films with other graphene-based composite films

Materials (wt/wt)	Nanosheet	Organic phase	Strain (%)	K (W m^{-1} K^{-1})	Refs.
rGO/CNF (40/60)	oriented	nanofiber	1.2	≈ 7	[S20]
rGO/CNF (50/50)	oriented	nanofiber	1	7.3	[S20]
rGO/CNF (30/70)	oriented	nanofiber	1.4	6.168	[S21]
rGO/CNF (50/50)	oriented	nanofiber	0.75	45	[S22]
rGO/PVDF (27.2/72.8)	oriented	polymer	1.5	19.5	[S23]
rGO/PDA (44.4/55.6)	oriented	polymer	6	13.42	[S24]
Graphene/PVDF (40/60)	oriented	polymer	2.5	20	[S25]
Graphene/PVDF (50/50)	oriented	polymer	1	28	[S25]
Graphene/PVDF (60/40)	oriented	polymer	1.25	41.64	[S25]
Graphene/PVDF (10/90)	random	polymer	–	1.47	[S26]
Graphene/PE (10/90)	random	polymer	–	1.84	[S26]

Graphene/PVDF (20/80)	random	polymer	–	2.06	[S27]
Graphene/PVDF (10/90)	random	polymer	12	15	[S28]
Graphene/PVDF (10/90)	random	polymer	–	2	[S29]
Graphene/PVDF (25/75)	random	polymer	–	10	[S29]
Graphene/ANF (20/80)	random	nanofiber	14	4	[S30]
**Graphene/ANF-X (40/60)**	**oriented**	**nanofiber network**	**18.6**	**48.2**	
**Graphene/ANF-X (50/50)**	**oriented**	**nanofiber network**	**15.3**	**76.9**	**This**
**Graphene/ANF-X (60/40)**	**oriented**	**nanofiber network**	**8.6**	**85.6**	**work**
**Graphene/ANF-X (70/30)**	**oriented**	**nanofiber network**	**5.5**	**91.3**	

rGO: reduced graphene oxide, CNF: cellulose nanofiber, PVDF: polyvinylidene fluoride, PDA: polydopamine, PE: polyethylene, ANF: aramid nanofiber, ANF-X: aramid nanofiber network.

Supplementary References

[S1] K. Liu, S.W. Chiang, B. Liang, C. Liang, Y. Sui et al., Exploration on the form factors of turbulence kinetic energy transfer for shear exfoliation of graphene. Nanotechnology **32**(26)**,** 265601 (2021). https://doi.org/10.1088/1361-6528/abef2d

[S2] C.S. Lee, S.J. Shim, T.H. Kim, Scalable preparation of low-defect graphene by urea-assisted liquid-phase shear exfoliation of graphite and its application in doxorubicin analysis. Nanomaterials **10**(2), 267 (2020). https://doi.org/10.3390/nano10020267

[S3] K. Zhang, J. Tang, J. Yuan, J. Li, Y. Sun et al., Production of few-layer graphene via enhanced high-pressure shear exfoliation in liquid for supercapacitor applications. ACS Appl. Nano Mater. **1**(6), 2877-2884 (2018). https://doi.org/10.1021/acsanm.8b00515

[S4] K.R. Paton, E. Varrla, C. Backes, R.J. Smith, U. Khan et al., Scalable production of large quantities of defect-free few-layer graphene by shear exfoliation in liquids. Nat. Mater. **13**(6), 624-630 (2014). https://doi.org/10.1038/nmat3944

[S5] J. Phiri, P. Gane, T.C. Maloney, High-concentration shear-exfoliated colloidal dispersion of surfactant–polymer-stabilized few-layer graphene sheets. J. Mater. Sci. **52**(13), 8321-8337 (2017). https://doi.org/10.1007/s10853-017-1049-y

[S6] T.S. Tran, S.J. Park, S.S. Yoo, T.R. Lee, T. Kim, High shear-induced exfoliation of graphite into high quality graphene by taylor-couette flow. RSC Adv. **6**(15), 12003-12008 (2016). https://doi.org/10.1039/c5ra22273g

[S7] X. Zhang, L. Wang, Q. Lu, D.L. Kaplan, Mass production of biocompatible graphene using silk nanofibers. ACS Appl. Mater. Interfaces **10**(27), 22924-22931 (2018). https://doi.org/10.1021/acsami.8b04777

[S8] K.R. Paton, J. Anderson, A.J. Pollard, T. Sainsbury, Production of few-layer graphene by microfluidization. Mater. Res. Express **4**(2), 025604 (2017). https://doi.org/10.1088/2053-1591/aa5b24

[S9] Y. Arao, Y. Mizuno, K. Araki, M. Kubouchi, Mass production of high-aspect-ratio few-layer-graphene by high-speed laminar flow. Carbon **102**, 330-338 (2016). https://doi.org/10.1016/j.carbon.2016.02.046

[S10] A.V. Tyurnina, I. Tzanakis, J. Morton, J. Mi, K. Porfyrakis et al., Ultrasonic exfoliation of graphene in water: a key parameter study. Carbon **168**, 737-747 (2020). https://doi.org/10.1016/j.carbon.2020.06.029

[S11] D. Shi, M. Yang, B. Chang, Z. Ai, K. Zhang et al., Ultrasonic-ball milling: a novel strategy to prepare large-size ultrathin 2D materials. Small **16**(13), e1906734 (2020).

https://doi.org/10.1002/small.201906734

[S12] M. Varenik, M.J. Green, O. Regev, Distinguishing self-assembled pyrene structures from exfoliated graphene. Langmuir **32**(41), 10699-10704 (2016). https://doi.org/10.1021/acs.langmuir.6b03379

[S13] R. Narayan, J. Lim, T. Jeon, D.J. Li, S.O. Kim, Perylene tetracarboxylate surfactant assisted liquid phase exfoliation of graphite into graphene nanosheets with facile re-dispersibility in aqueous/organic polar solvents. Carbon **119**, 555-568 (2017). https://doi.org/10.1016/j.carbon.2017.04.071

[S14] Q. Wan, H. Wang, S. Li, J. Wang, Efficient liquid-phase exfoliation of few-layer graphene in aqueous 1, 1, 3, 3-tetramethylurea solution. J. Colloid Interf. Sci. **526**, 167-173 (2018). https://doi.org/10.1016/j.jcis.2018.04.110

[S15] Y. Hernandez, V. Nicolosi, M. Lotya, F.M. Blighe, Z. Sun et al., High-yield production of graphene by liquid-phase exfoliation of graphite. Nat. Nanotechnol. **3**(9), 563-568 (2008). https://doi.org/10.1038/nnano.2008.215

[S16] A. Islam, B. Mukherjee, K.K. Pandey, A.K. Keshri, Ultra-fast, chemical-free, mass production of high quality exfoliated graphene. ACS Nano **15**(1), 1775-1784 (2021). https://doi.org/10.1021/acsnano.0c09451

[S17] C. Teng, D. Xie, J. Wang, Z. Yang, G. Ren et al., Ultrahigh conductive graphene paper based on ball-milling exfoliated graphene. Adv. Funct. Mater. **27**(20), 1700240 (2017). https://doi.org/10.1002/adfm.201700240

[S18] Ö. Güler, A. Sönmez, The effect of liquid media on the efficiency of graphene production by liquid-phase exfoliation from micromechanically pre-exfoliated graphite. J. Electron. Mater. **49**(9), 5335-5345 (2020). https://doi.org/10.1007/s11664-020-08257-w

[S19] J. Xu, X. Zhao, F. Liu, L. Jin, G. Chen, Preparation of graphene via wet ball milling and in situ reversible modification with the diels-alder reaction. New J. Chem. **44**(4), 1236-1244 (2020). https://doi.org/10.1039/c9nj05309c

[S20] W. Yang, Z. Zhao, K. Wu, R. Huang, T. Liu et al., Ultrathin flexible reduced graphene oxide/cellulose nanofiber composite films with strongly anisotropic thermal conductivity and efficient electromagnetic interference shielding. J. Mater. Chem. C **5**(15), 3748-3756 (2017). https://doi.org/10.1039/c7tc00400a

[S21] N. Song, D. Jiao, P. Ding, S. Cui, S. Tang et al., Anisotropic thermally conductive flexible films based on nanofibrillated cellulose and aligned graphene nanosheets. J. Mater. Chem. C **4**(2), 305-314 (2016). https://doi.org/10.1039/c5tc02194d

[S22] G. Li, X. Tian, X. Xu, C. Zhou, J. Wu et al., Fabrication of robust and highly thermally conductive nanofibrillated cellulose/graphite nanoplatelets composite papers. Compos. Sci. Technol. **138**, 179-185 (2017). https://doi.org/10.1016/j.compscitech.2016.12.001

[S23] P. Kumar, S. Yu, F. Shahzad, S.M. Hong, Y.H. Kim et al., Ultrahigh electrically and thermally conductive self-aligned graphene/polymer composites using large-area reduced graphene oxides. Carbon **101**, 120-128 (2016). https://doi.org/10.1016/j.carbon.2016.01.088

[S24] F. Luo, K. Wu, J. Shi, X. Du, X. Li et al., Green reduction of graphene oxide by polydopamine to a construct flexible film: superior flame retardancy and high thermal conductivity. J. Mater. Chem. A **5**(35), 18542-18550 (2017). https://doi.org/10.1039/c7ta04740a

[S25] T. Hu, Y. Song, J. Di, D. Xie, C. Teng, Highly thermally conductive layered polymer composite from solvent-exfoliated pristine graphene. Carbon **140**, 596-602 (2018). https://doi.org/10.1016/j.carbon.2018.09.026

[S26] F.E. Alam, W. Dai, M. Yang, S. Du, X. Li et al., In situ formation of a cellular graphene framework in thermoplastic composites leading to superior thermal conductivity. J. Mater. Chem. A **5**(13), 6164-6169 (2017). https://doi.org/10.1039/c7ta00750g

[S27] Y. Cao, M. Liang, Z. Liu, Y. Wu, X. Xiong et al., Enhanced thermal conductivity for poly(vinylidene fluoride) composites with nano-carbon fillers. RSC Adv. **6**(72), 68357-68362 (2016). https://doi.org/10.1039/c6ra11178e

[S28] A.A. Tarhini, A.R.T. Bagha, Graphene-based polymer composite films with enhanced mechanical properties and ultra-high in-plane thermal conductivity. Compos. Sci. Technol. **184**, 107797 (2019). https://doi.org/10.1016/j.compscitech.2019.107797

[S29] H. Jung, S. Yu, N.S. Bae, S.M. Cho, R.H. Kim et al., High through-plane thermal conduction of graphene nanoflake filled polymer composites melt-processed in an L-shape kinked tube. ACS Appl. Mater. Interfaces **7**(28), 15256-15262 (2015). https://doi.org/10.1021/acsami.5b02681

[S30] L.H. Zhao, Y.F. Jin, Z.G. Wang, J.W. Ren, L.C. Jia et al., Highly thermally conductive fluorinated graphene/aramid nanofiber films with superior mechanical properties and thermostability. Ind. Eng. Chem. Res. **60**(23), 8451-8459 (2021). https://doi.org/10.1021/acs.iecr.1c01260
